# Improving the use of research evidence in guideline development: 14. Reporting guidelines

**DOI:** 10.1186/1478-4505-4-26

**Published:** 2006-12-08

**Authors:** Andrew D Oxman, Holger J Schünemann, Atle Fretheim

**Affiliations:** 1 Norwegian Knowledge Centre for the Health Services, P.O. Box 7004, St. Olavs plass, N-0130 Oslo, Norway; 2 INFORMA, S.C. Epidemiologia, Istitituto Regina Elena, Via Elio Chianesi 53, 00144 Rome, Italy; 3 Norwegian Knowledge Centre for the Health Services, P.O. Box 7004, St. Olavs plass, N-0130 Oslo, Norway

## Abstract

**Background:**

The World Health Organization (WHO), like many other organisations around the world, has recognised the need to use more rigorous processes to ensure that health care recommendations are informed by the best available research evidence. This is the 14^th ^of a series of 16 reviews that have been prepared as background for advice from the WHO Advisory Committee on Health Research to WHO on how to achieve this.

**Objectives:**

We reviewed the literature on reporting guidelines and recommendations.

**Methods:**

We searched PubMed and three databases of methodological studies for existing systematic reviews and relevant methodological research. We did not conduct systematic reviews ourselves. Our conclusions are based on the available evidence, consideration of what WHO and other organisations are doing and logical arguments.

**Key questions and answers:**

There is little empirical evidence that addresses these questions. Our answers are based on logical arguments and standards put forward by other groups.

What standard types of recommendations or reports should WHO use?

• WHO should develop standard formats for reporting recommendations to facilitate recognition and use by decision makers for whom the recommendations are intended, and to ensure that all the information needed to judge the quality of a guideline, determine its applicability and, if needed, adapt it, is reported.

• WHO should develop standard formats for full systematically developed guidelines that are sponsored by WHO, rapid assessments, and guidelines that are endorsed by WHO.

• All three formats should include the same information as full guidelines, indicating explicitly what the group preparing the guideline did not do, as well as the methods that were used.

• These formats should be used across clinical, public health and health systems recommendations.

How should recommendations be formulated and reported?

• Reports should be structured, using headings that correspond to those suggested by the Conference on Guideline Standardization or similar headings.

• The quality of evidence and strength of recommendations should be reported explicitly using a standard approach.

• The way in which recommendations are formulated should be adapted to the specific characteristics of a specific guideline.

• Urgent attention should be given to developing a template that provides decision makers with the relevant global evidence that is needed to inform a decision and offers practical methods for incorporating the context specific evidence and judgements that are needed.

## Background

The World Health Organization (WHO), like many other organisations around the world, has recognised the need to use more rigorous processes to ensure that health care recommendations are informed by the best available research evidence. This is the 14^th ^of a series of 16 reviews that have been prepared as background for advice from the WHO Advisory Committee on Health Research to WHO on how to achieve this.

Guidelines are formal advisory statements that should be robust enough to meet the unique circumstances and constraints of the specific situation to which they are being applied [1]. The basic nature and intent of guidelines have been variously labelled as guidance, guides, guiding principles, recommendations, policies, protocols, best practice, algorithms, consensus statements, expert committee recommendations, integrated care pathways, manuals, tool kits, handbooks, model lists, technical updates and principles [1,2]. Whatever they are called, rigorously developed guidelines, can translate complicated research findings into actionable recommendations. They are an important step in moving from research to action and ensuring that the best available research evidence informs decisions and actions [3]. However, for users of guidelines to be able to apply criteria to assess whether guidelines have been rigorously developed and are likely to be valid and applicable [3-6], the information needed to make these judgements must be reported [7]. Unfortunately, critical information is often absent from published guidelines [5,7,8].

In this paper we address the following questions:

• What standard types of recommendations or reports should WHO use?

• How should recommendations be formulated and reported?

We address questions about reporting systematic reviews [9] and dissemination [10] in other papers in this series.

## What WHO is doing now

Although the Guidelines for WHO Guidelines recommends "that a uniform, readily-recognizable printing format be developed for WHO guidelines," there are, as yet, no standard formats for WHO policies, recommendations or guidelines. A survey of WHO guidelines published in 2005 found that WHO publishes a large number of recommendations of many different types, in many different formats [2], and a review of WHO documents did not find any standards for reporting WHO recommendations [11].

## What other organisations are doing

In a recent survey of organisations that produce clinical practice guidelines, all 31 organisations that responded (response rate 86%), and 46 of 57 (81%) of units that support the use of research evidence by governments in developing health policy, reported producing full versions of guidelines with references and notes [12]. Several organisations use different formats for different types of recommendations, and a majority produce different versions of guidelines, such as executive summaries, summaries of take-home messages, separate versions for different target users, and tools for application (e.g., algorithms or flow charts). Many guideline producers have standard formats that they use and some organisations, such as the U.S. National Guidelines Clearing House, have developed standard formats for reporting guidelines produced by other organisations [13].

The UK National Center for Health and Clinical Excellence (NICE) states that recommendations should be clear and concise, but should contain sufficient information that they can be understood without reference to other supporting material [14]. This is particularly important where recommendations are published in isolation from the background details in the full guideline. Any terminology included in the recommendations therefore needs to be clearly defined and unambiguous.

## Methods

The methods used to prepare this review are described in the introduction to this series [15]. Briefly, the key questions addressed in this paper were vetted amongst the authors and the ACHR Subcommittee on the Use of Research Evidence (SURE). We did not conduct a full systematic review. We searched PubMed and three databases of methodological studies (the Cochrane Methodology Register [16], the US National Guideline Clearinghouse [17], and the Guidelines International Network [18]) for existing systematic reviews and relevant methodological research that address these questions. The answers to the questions are our conclusions based on the available evidence, consideration of what WHO and other organisations are doing, and logical arguments.

For this review we searched PubMed using [the MeSH terms 'Documentation/standards' and 'Practice Guidelines/standards'] and related articles; the Cochrane Methodology Register using [the key words 'CMR: Review methodology – applicability & recommendations' and the text words (format or reporting)] and ['Levels of evidence and strength of recommendations']; the National Guidelines Clearinghouse annotated bibliography using the terms format, reporting and structure; and checked the reference lists of retrieved articles.

## Findings

### What standard types of recommendations or reports should WHO use?

Given the wide variety of different types of recommendations that are made by WHO, there is likely to be a need for several standard types of recommendations. Systematically developed clinical practice guidelines can take 18 months or more and as many as 15 meetings [19]. Systematically developed public health guidelines also require substantial resources and time [20]. Given the time and resources required to produce guidelines, many organisations, particularly HTA organisations, have developed rapid assessment processes [21-25]. There is variation in the scope, methods, time to complete assessments, and the formats used to report rapid assessments. Another type of recommendation or guideline that is receiving increasing attention, also because of the resources and time required to develop guidelines systematically, are guidelines developed by other organisations that have been adapted or endorsed [26-28]. Another approach being taken in several countries is to create databases or clearinghouses of clinical practice guidelines with the aim of facilitating their evaluation and adaptation for local use by health care organizations [29].

Systematically developed clinical recommendations, public health recommendations, and health systems recommendations all require similar processes to ensure their quality. Decision makers also require similar types of information to be able to critically appraise whether guidelines have been rigorously developed and are likely to be valid [3,4,7,20,30,31].

### How should recommendations be formulated and reported?

The Conference on Guideline Standardization (COGS) used a two-stage modified Delphi process to develop standards for reporting clinical practice guidelines [7]. Representatives of 22 organisations active in guideline development reviewed the proposed items and commented favourably. The items were consolidated into 18 topics (Table 1) to create the COGS checklist, which provides a framework to support comprehensive documentation of guidelines. While it is possible that some guideline developers may not include content for every item, it is suggested that they should address explicitly whether the guideline development team considered that item.

**Table 1:** The COGS checklist for reporting clinical practice guidelines (from Shiffman et al. [7])*

**Topic**	**Description**
1. Overview material	Provide a structured abstract that includes the guideline's release date, status (original, revised, updated), and print and electronic sources.
2. Focus	Describe the primary disease/condition and intervention/service/technology that the guideline addresses. Indicate any alternative preventive, diagnostic or therapeutic interventions that were considered during development.
3. Goal	Describe the goal that following the guideline is expected to achieve, including the rationale for development of a guideline on this topic.
4. Users/setting	Describe the intended users of the guideline (e.g., provider types, patients) and the settings in which the guideline is intended to be used.
5. Target population	Describe the patient population eligible for guideline recommendations and list any exclusion criteria.
6. Developer	Identify the organization(s) responsible for guideline development and the names/credentials/potential conflicts of interest of individuals involved in the guideline's development.
7. Funding sources/sponsor	Identify the funding source/sponsor and describe its role in developing and/or reporting the guideline. Disclose potential conflict of interest.
8. Evidence collection	Describe the methods used to search the scientific literature, including the range of dates and databases searched, and criteria applied to filter the retrieved evidence.
9. Recommendation grading criteria	Describe the criteria used to rate the quality of evidence that supports the recommendations and the system for describing the strength of the recommendations. Recommendation strength communicates the importance of adherence to a recommendation and is based on both the quality of the evidence and the magnitude of anticipated benefits or harms.
10. Method for synthesizing evidence	Describe how evidence was used to create recommendations, e.g., evidence tables, meta-analysis, decision analysis.
11. Prerelease review	Describe how the guideline developer reviewed and/or tested the guidelines prior to release.
12. Update plan	State whether or not there is a plan to update the guideline and, if applicable, an expiration date for this version of the guideline.
13. Definitions	Define unfamiliar terms and those critical to correct application of the guideline that might be subject to misinterpretation.
14. Recommendations and rationale	State the recommended action precisely and the specific circumstances under which to perform it. Justify each recommendation by describing the linkage between the recommendation and its supporting evidence. Indicate the quality of evidence and the recommendation strength, based on the criteria described in 9.
15. Potential benefits and harms	Describe anticipated benefits and potential risks associated with implementation of guideline recommendations.
16. Patient preferences	Describe the role of patient preferences when a recommendation involves a substantial element of personal choice or values.
17. Algorithm	Provide (when appropriate) a graphical description of the stages and decisions in clinical care described by the guideline.
18. Implementation considerations	Describe anticipated barriers to application of the recommendations. Provide reference to any auxiliary documents for providers or patients that are intended to facilitate implementation. Suggest review criteria for measuring changes in care when the guideline is implemented.

*COGS = Conference on Guideline Standardization.

While many organisations have their own standard formats for reporting guidelines, this is the only consensus standard for reporting guidelines across organisations. We have, however, summarised the key items included in guidelines for guidelines, which provides the basis for a similar, but more comprehensive checklist for conducting or reporting guidelines [32]. In addition, there are a number of instruments for evaluating clinical practice guidelines that can also be used as checklists for reporting [1,4-6,33]. The content used in the National Guideline Clearing house also represents a standard for reporting imposed on organisations that want their guidelines included in that database [13]. It includes 52 items under the following headings: scope, methodology – including rating scheme and cost analysis, recommendations, evidence supporting the recommendations, benefits/harms of implementing the recommendations, contraindications, qualifying statements, implementation of the guideline, Institute of Medicine (IOM) national healthcare quality report categories, identifying information and availability, and disclaimer; in addition to indexing attributes.

Similarly, some journals have standard formats for reporting clinical practice guidelines, including structured abstracts with the following headings [34]:

#### Objective

a succinct statement of the objective of the guideline, including the targeted health problem, the targeted patients and providers, and the main reason for developing recommendations concerning this problem for this population.

#### Options

principal practice options that were considered in formulating the guideline.

#### Outcomes

significant health and economic outcomes identified as potential consequences of the practice options.

#### Evidence

methods used to gather, select, and synthesize evidence, and the date of the most recent evidence obtained.

#### Values

persons and methods used to assign values (relative importance) to potential outcomes of alternative practice options.

#### Benefits, harms, and costs

the type and magnitude of the main benefits, harms, and costs that are expected to result from guideline implementation.

#### Recommendations

a brief and specific list of key recommendations.

#### Validation

the results of any external review, comparison with guidelines developed by other groups, or clinical testing of guideline use.

#### Sponsors

key persons or groups that developed, funded, or endorsed the guideline.

While many organisations have standards for how recommendations are formulated, we are not aware of any consensus standards for how recommendations should be formulated. Most guidelines development groups now grade the quality of evidence and the strength of recommendations, but a variety of different grading systems are used [12,35].

Shekelle and colleagues evaluated the effect of different levels of specificity of recommendations on clinicians test ordering behaviour using clinical vignettes [36]. They found that clinicians receiving the non-specific recommendations ordered fewer indicated tests for appropriate clinical vignettes than did physicians receiving specific recommendations. Furthermore, compared to physicians receiving non-specific guidelines, physicians receiving specific guidelines ordered significantly more appropriate tests for corresponding vignettes and significantly fewer tests for inappropriate vignettes. The authors concluded that the clarity and clinical applicability of a guideline might be important attributes that contribute to the effects of practice guidelines. We did not find any other comparisons of different ways of formulating recommendations, and it is likely that the way in which recommendations are formulated may need to be adapted to the specific characteristics of a guideline. However, there is a consensus that recommended actions should be stated precisely [7].

Both checklists for evaluating guidelines and for reporting guidelines include items that may be dependent on the specific setting in which a guideline is developed or used. There are a number of international organisations, including WHO, that develop guidelines that are intended to be used in a variety of settings around the world. We did not find any published papers that addressed methods for taking into account setting specific factors in international guidelines, although several groups are working on methods for adapting guidelines developed in one setting for use in another [27,28,37-39].

## Discussion

While the content of WHO guidelines, recommendations and policies will vary, depending on the topic, it would be desirable to have standard formats across different topics to facilitate recognition and use by decision makers and ensure that all the information needed to judge the quality of a guideline, determine its applicability and, if needed, adapt it is reported. There is likely a need for at least three standard formats: full systematically developed guidelines that are sponsored by WHO, rapid assessments, and guidelines that are endorsed by WHO. Standards such as those advocated by COGS should form the basis for developing a uniform format for full guidelines developed by WHO. Although the COGS standards were developed for clinical practice guidelines, the same considerations are relevant to public health and health systems recommendations. Further consideration is needed regarding the inclusion of additional items that need to be considered in WHO guidelines, such as applicability to different settings, equity, and scaling up. In particular, work is needed to develop a template for decision-making frameworks when different conditions are likely to lead to different decisions in different settings [40,41]. In addition, different versions of guidelines should be developed for different target audiences, including a structured executive summary and key messages [31,34].

A different format for rapid assessments would help to distinguish these from full guidelines and could be designed to reduce the work and time necessary to complete a report. Rapid assessments should, nonetheless, include the same information as full guidelines, indicating explicitly what the group preparing the guideline did not do, as well as the methods that were used.

WHO has limited resources and capacity for developing guidelines. At the same time, low and middle-income (LMIC) countries also have limited resources and capacity, and a core function of WHO is to provide its member states, particularly LMIC, with technical advice that is informed by the best available research evidence. Through collaborating with other organisations and establishing standards for reporting, and possibly endorsing guidelines developed by other organisations, WHO may be able to expand its potential for supporting access to guidelines that are appropriate for LMIC or can easily be adapted to conditions in LMIC.

Given WHO's role as the world's leading public health agency and its mandate to provide evidence-informed guidance, it is of major importance for WHO to address and develop methods for formulating and reporting recommendations that are developed internationally, but need to be adapted and implemented in specific settings.

## Further work

As part of the methodological work that is needed to addresses the challenges of developing international guidelines, specific attention should be given to the development of standard templates that provide decision makers with the relevant global evidence that is needed to inform a decision and offers practical methods for incorporating the context specific evidence and judgements that are needed [40,41].

In addition to ensuring that standard formats are used for WHO guidelines to ensure complete reporting, attention should be paid to ensuring that the format that is used, and derivative versions, are understandable and useful to the intended target audiences.

## Competing interests

ADO and AF work for the Norwegian Knowledge Centre forthe Health Services, an agency funded by the Norwegian government that produces systematic reviews and health technology assessments. All three authors are contributors to the Cochrane Collaboration. ADO and HJS are members of the GRADE Working Group. HJS is documents editor and chair of the documents development and implementation committee for the American Thoracic Society and senior editor of the American College of Chest Physicians' Antithrombotic and Thrombolytic Therapy Guidelines.

## Authors' contributions

ADO prepared the first draft of this review. HJS and AF contributed to drafting and revising it.
